# Supplementary figures and images for: The Effects of Salinity on the Survival, Growth, and Eco-Physiological Parameters of Juvenile Sea Urchin Diadema setosum
Source: Animals (Basel). 2025 Aug 21;15(16):2462. doi: 10.3390/ani15162462 (PMC12542878; doi:10.3390/ani15162462)

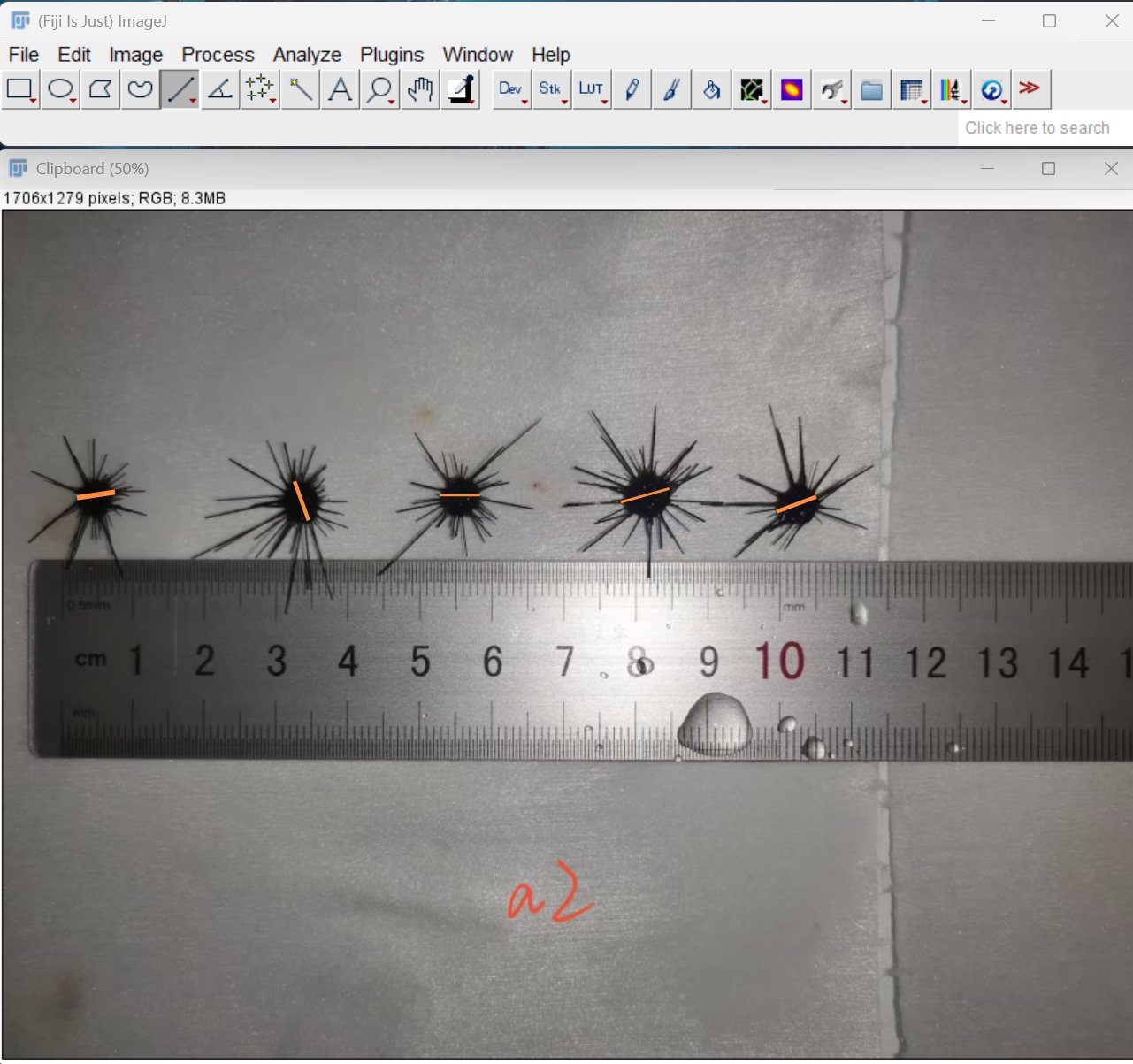

Supplement: Supplementary file 1 [file animals-15-02462-s001.zip › Supplementary Material/Figure S1.jpg]

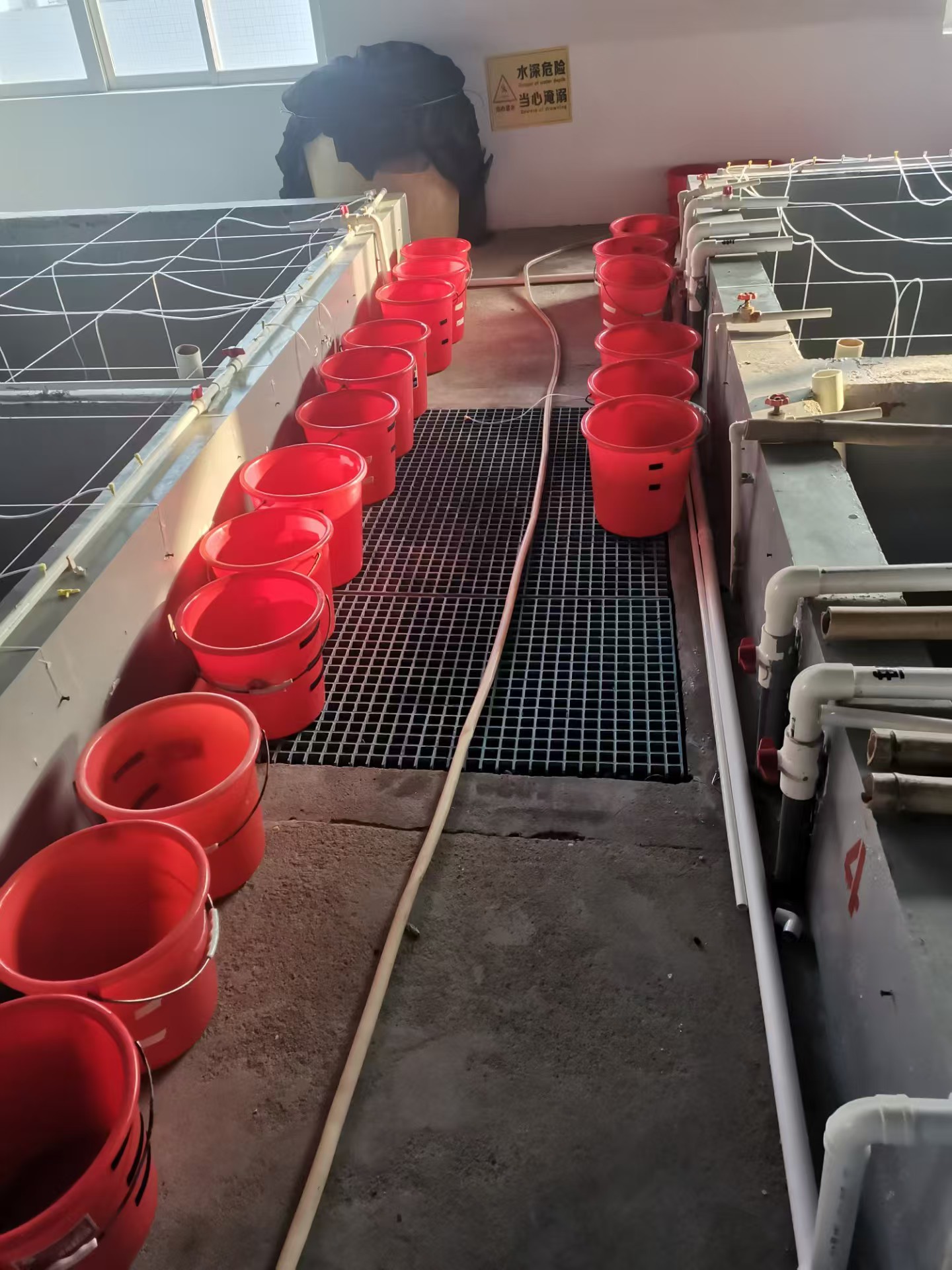

Supplement: Supplementary file 1 [file animals-15-02462-s001.zip › Supplementary Material/Figure S3.jpg]

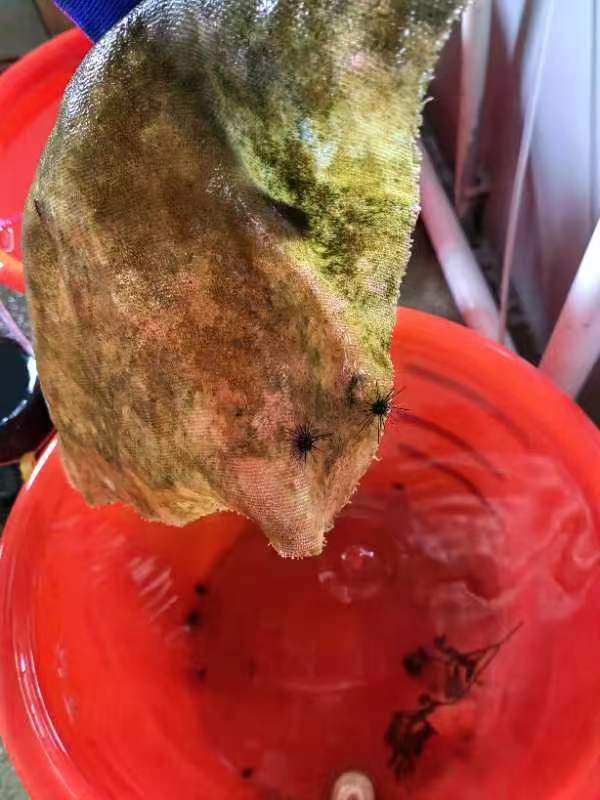

Supplement: Supplementary file 1 [file animals-15-02462-s001.zip › Supplementary Material/Figure S4.jpg]

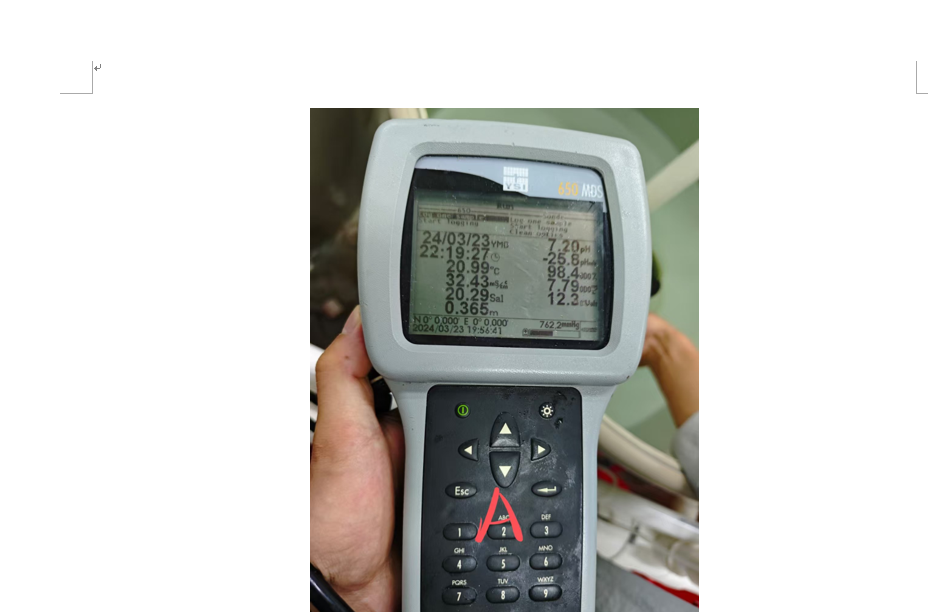

Supplement: Supplementary file 1 [file animals-15-02462-s001.zip › Supplementary Material/Figure S5.png]

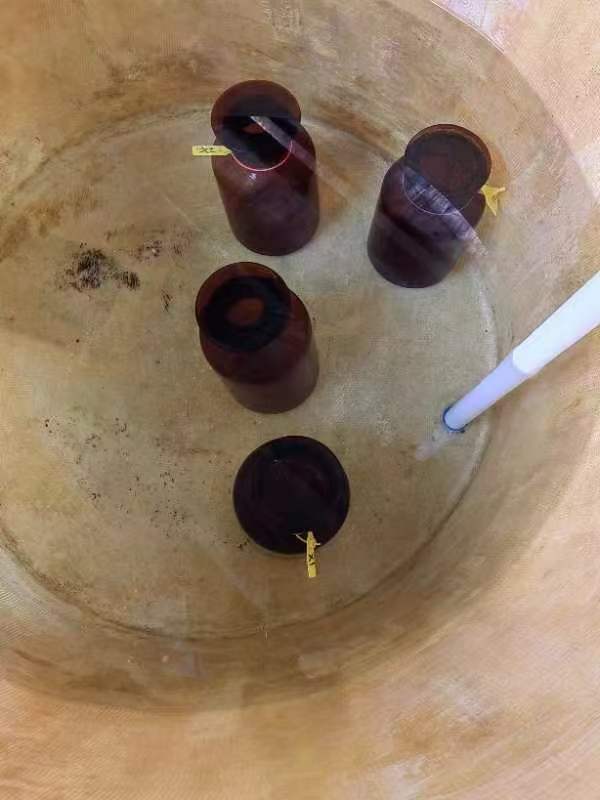

Supplement: Supplementary file 1 [file animals-15-02462-s001.zip › Supplementary Material/Figure S6.jpg]

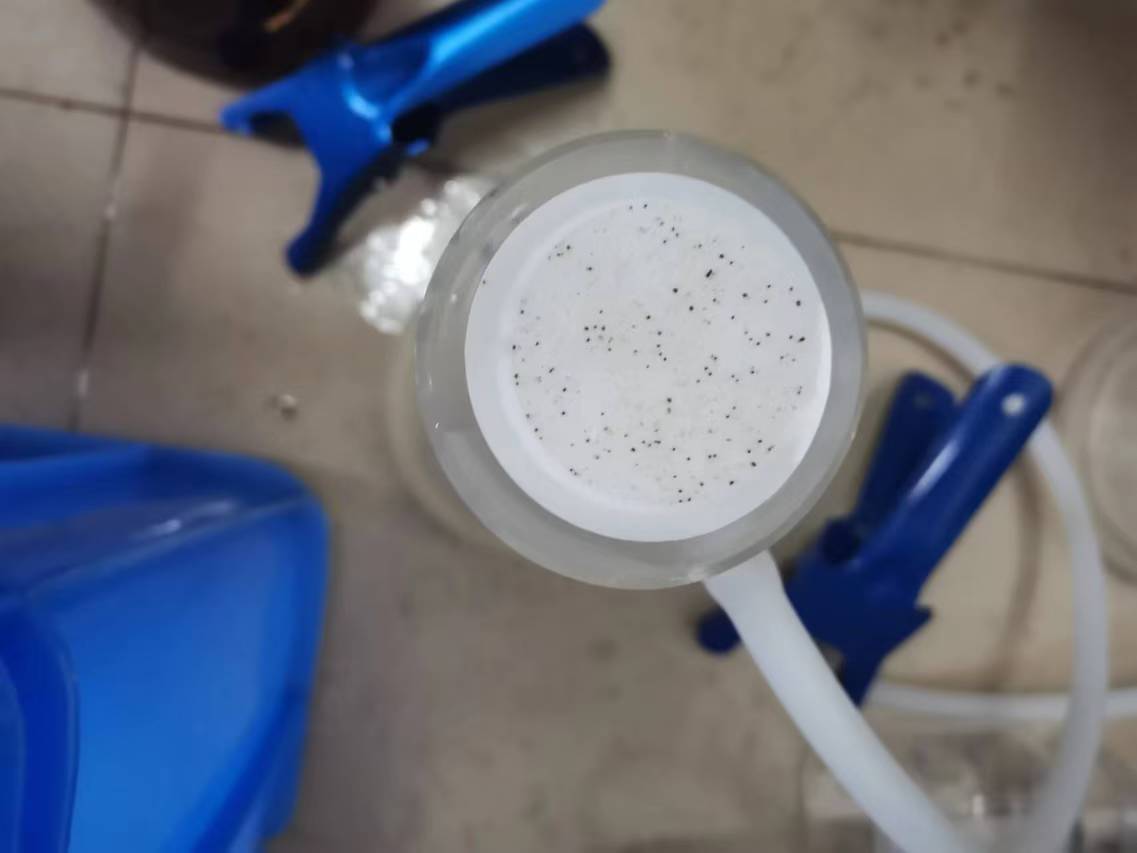

Supplement: Supplementary file 1 [file animals-15-02462-s001.zip › Supplementary Material/Figure S7.jpg]

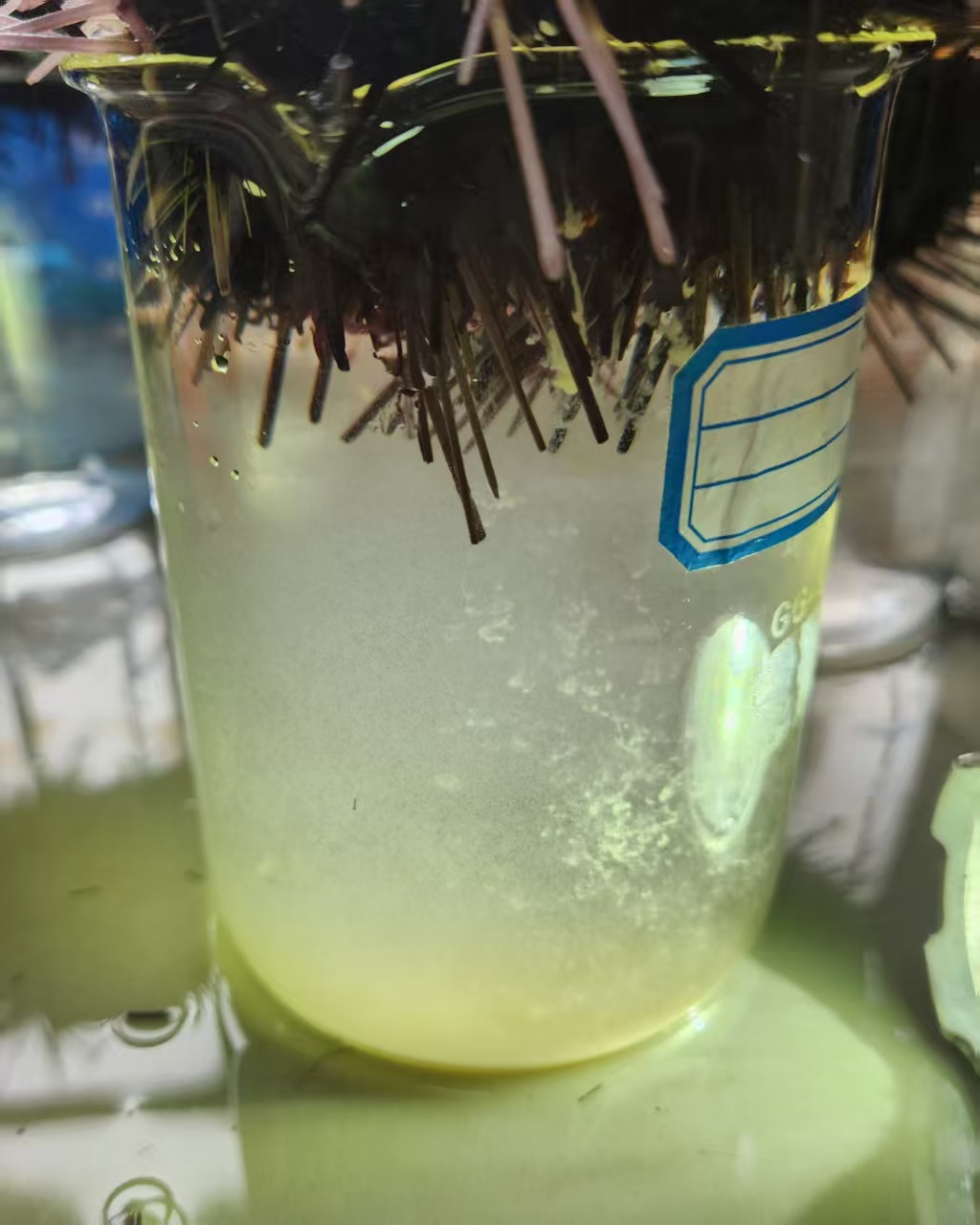

Supplement: Supplementary file 1 [file animals-15-02462-s001.zip › Supplementary Material/Figure S2.jpg]
